# Hunter selection for larger and older male bobcats affects annual harvest demography

**DOI:** 10.1098/rsos.180668

**Published:** 2018-10-10

**Authors:** Maximilian L. Allen, Nathan M. Roberts, Timothy R. Van Deelen

**Affiliations:** 1 Illinois Natural History Survey, 1816 S. Oak Street, Champaign, IL 61820, USA; 2 Wisconsin Department of Natural Resources, 107 Sutliff Avenue, Rhinelander, WI 54501, USA; 3 Department of Forest and Wildlife Ecology, University of Wisconsin, 1630 Linden Drive, Madison, WI 53706, USA

**Keywords:** bobcat, hunting methods, *Lynx rufus*, selection, trophy hunting, wildlife demography

## Abstract

Wildlife researchers often rely on demographic data collected from harvested animals to estimate population dynamics. But demographic data from harvested animals may be non-representative if hunters/trappers have the ability and motivation to preferentially select for certain physical traits. Hunter preference is well demonstrated for ungulates, but less so for other wildlife species such as furbearers. We used data from bobcats harvested in Wisconsin (1983–2014) to determine if harvest method and demographics (mass, male:female sex ratio and age) have changed over time, and if bobcat hunters/trappers exhibited selection. Each trait of harvested bobcats that we tested changed over time, and because these selected traits were interrelated, we inferred that harvest selection for larger size biased harvests in favour of older, male bobcats. The selection of older, male bobcats appears primarily driven by hound hunters (hereafter hunters) compared to trappers, with hunters more frequently creating taxidermy mounts from their harvested bobcats. We found an increase in the proportion of bobcats that were harvested by hunting compared to trapping over time, and this was associated with increased selectivity and substantial changes in the characteristics of harvested bobcats. Selection by hunters may bias population models that are based on the demography of harvested bobcats, and accounting for biases that may occur, including from different harvest methods, is critical when using harvest-dependent data.

## 1. Introduction

Quantifying and estimating wildlife populations is a foundation of wildlife management and research. However, many carnivore species are cryptic [1,2], leading to innate difficulties in estimating population size and trend [1,3]. Given these difficulties, researchers often rely on demographic data collected from harvested animals to estimate population parameters such as survival, recruitment and population growth (e.g. [4]). The observation processes used by biologists, however, are not always accurate measures of the biological processes underlying changes to populations [5,6]. Harvest dynamics can change over the course of decades with changes in management and regulation, and it is important to understand how selection can affect the observation processes that researchers use, particularly when harvest data are used to determine population size and trends (e.g. [6]).

Harvest itself can affect the sex and age structure of the population [6–9], especially in cases of overharvest or when hunters exhibit selection for traits related to sex and age [5,8,10]. Male animals in populations are more often selected for trophy traits [5,6,11,12], or are more vulnerable to harvest due to larger movement patterns and riskier behaviour [9,12,13]. Selection for certain traits is most frequently observed in ungulates [5,11], but does not always occur and can be mitigated by subsistence or other types of hunting [12]. For example, in roe deer, foreign hunters selected for trophy animals compared to local hunters [11], primarily because foreign hunters often paid large fees for hunting and were able to hunt earlier and in prime locations [11]. In brown bears (*Ursus arctos*) in Scandinavia, however, there was no difference in selectivity by the method of hunting [9], and selectivity among non-ungulates needs to be better understood (e.g. [12]).

Carnivore researchers need to understand variation in selection and sampling from different hunting methods, and their effects on population parameters and estimation techniques. Furbearers, such as bobcats (*Lynx rufus*), gray foxes (*Urocyon cinereoargenteus*) and raccoons (*Procyon lotor*), traditionally have been harvested to sell their fur [14,15]. Trapper effort and harvest of furbearers have often correlated to pelt price [14,16,17], and selection for furbearers historically has reflected the phenological quality of the pelt and, for some species, avoiding young of the year. However, trap type, seasonal restrictions, and weather can affect the capture and annual harvest of furbearers [3,15]. For example, beaver (*Castor canadensis*) continue to grow until 4 or 5 years of age [18], so that during any given trapping season their sizes may range from 5 kg to greater than 30 kg [18], and beaver traps can be made to select larger animals. Similarly, weather can impact access to trapping lands (e.g. some roads are not maintained during the winter, some areas might only be accessible with snowmobiles or skis after snowfall) [19].

Bobcats can be harvested by trapping, calling (using auditory lures to bring the bobcat within range of the waiting hunter), or by hunters who use hounds to find, pursue and tree them (hereafter ‘hunters’). These harvest methods are inherently different and could lead to variation in the observation processes of harvest-based models. Hunters can exhibit selection at multiple points, first when selecting a track to follow and second when choosing whether to harvest a treed individual or pursue a different individual. Trappers, however, have an animal caught in a trap, which can be difficult to release, and may be less likely to release the animal and attempt to catch another.

We studied a bobcat population in Wisconsin, USA, where bobcats are distributed and harvested throughout the state. Bobcats are a cryptic, solitary felid, whose populations are spatially dispersed and tend to have a higher proportion of resident females relative to males [20–22]. Survival for males tends to be lower than females in hunted populations [23], but is similar in un-hunted populations [24]. The population in Wisconsin has likely increased coincident with a general increase in bobcat populations in the continental United States in recent decades [25,26]. Changes in management by the Wisconsin Department of Natural Resources (WDNR) have affected the bobcat harvest in the state over time. A bounty system for bobcats was implemented in the state in 1867, and continued until 1964, with mandatory registration of harvested bobcats beginning in 1973 [27]. In 1980, harvest was restricted to the northern 1/3 of the state north with a limit of 1 bobcat per harvest permit, and then shifted to the current quota system in 1992 [27]. The number of available bobcat harvest permits in Wisconsin has greatly decreased over time, which may have potentially increased the perceived value of a harvested bobcat beyond its pelt price [15]. Increases in hunter skill and value of the harvested animal could potentially lead to increased selectivity for animals with specific traits (i.e. trophy animals) that could bias sampling of harvested animals from the population. Understanding changing trends in harvest, hunting technique and harvest demography is important in estimating population parameters.

We used data from bobcats harvested in Wisconsin (1983–2014) to understand if demographics of harvested bobcats have changed over time, as well as if hunters/trappers are exhibiting selection for bobcats with certain traits. We first performed a baseline analysis to determine if the mass of harvested bobcats varied significantly by sex and age. We then had four objectives: (1) Determine if there has been a changing proportion in the method of harvest between trappers and hunters over time. We hypothesized that an increasing bobcat population and increasing value of harvest permits leads to an increase in skill and investment by hunters leading to a greater proportion of bobcats harvested by hunters than trappers. (2) Determine whether aspects of bobcat demographics (mass, male:female sex ratio and age) changed over time. We hypothesized that with an increasing bobcat population and increasing proportion of hunters compared to trappers we would see an increase in each of these demographic metrics over time. (3) Determine if there was selectivity among hunters and trappers, and whether selectivity increased over time with an increasing population. We hypothesized that hunters would select for bobcats that were (a) male, (b) larger and (c) older, and that this selectivity would increase with increasing bobcat populations over time that allows for increased selectivity among individual bobcats to harvest; while trappers would have less opportunities for selection due to their method. (4) Determine if the final disposition of harvested bobcats vary by method, hypothesizing that animals with trophy characteristics may be made into taxidermy mounts and would more likely be created by hunters than trappers.

## 2. Material and methods

### 2.1. Data collection

**Figure 1.**
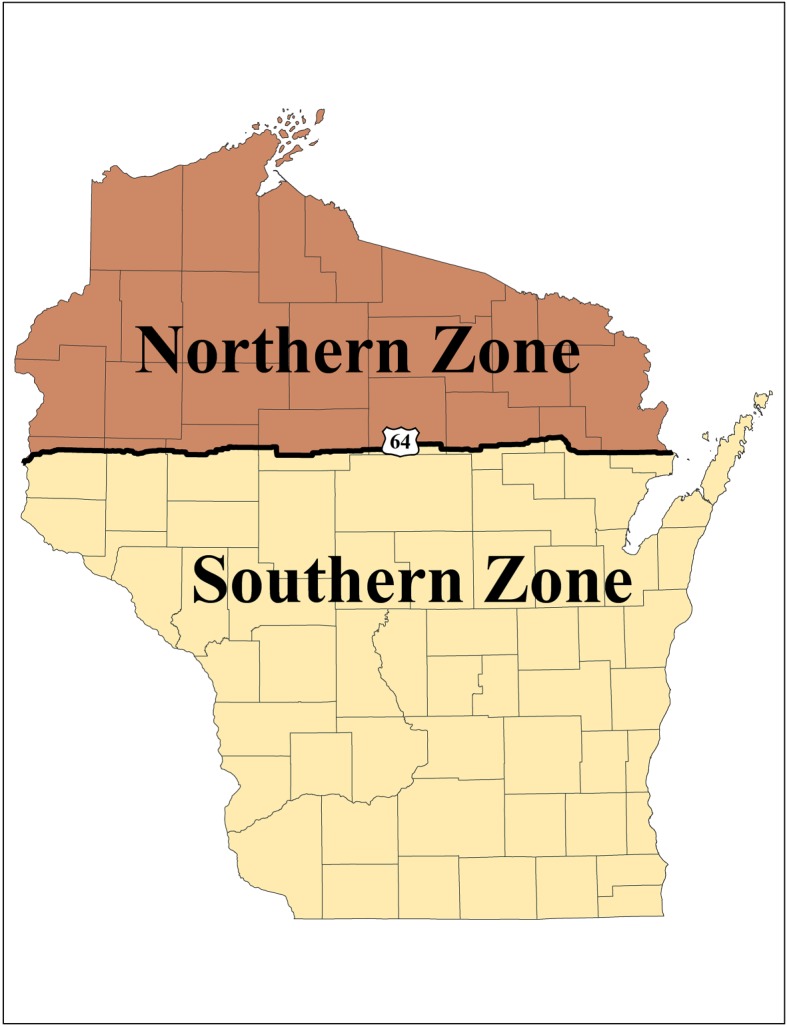
Map of the study area showing the bobcat zones in Wisconsin delineated by Highway 64. The southern bobcat zone was added in 2014.

We used bobcat harvest data from 1973 to 2014 in Wisconsin's northern zone (figure 1), because harvest for bobcats in Wisconsin's southern zone only began in 2014. Since 1973, the WDNR has required bobcat hunters/trappers to register every harvested bobcat with WDNR law enforcement personnel within 5 days after the month of harvest. The harvest of bobcats is regulated by the WDNR, and the use of harvest data did not entail any research ethic permits, animal research permits or fieldwork permits.

Since 1983, the WDNR has required bobcat hunters/trappers to submit the skinned carcass of harvested bobcats in order to obtain age, sex, mass (only collected until 1999), pregnancy rates and litter size. Bobcat teeth were submitted to Matson's Laboratory (Milltown, MT, USA) for ageing through analysis of cementum annuli [28]. Bobcat teeth were aged to year when possible (73.8%), and bobcats with unknown ages were not included in analyses. Most of our analyses focused on 5754 bobcats harvested in Wisconsin from 1983 to 2014 for which we had complete demographic data (age, sex), but data for mass was only available from 1983 to 2014. Demographic data do not distinguish between state and tribal harvest, and we therefore use this lumped data without attempting to demarcate these sources.

We used the annual bobcat population estimate from the WDNR [26] as our values for the bobcat population. The WDNR estimate is a sex-age-kill model based on a Minnesota Furbearer Population Model and is integrated with an index of winter bobcat track counts [26]. The WDNR population estimate is not independent of the sex and age data we analyse but is the best proxy for population abundance available for bobcats in Wisconsin. The WDNR sent surveys annually from 1993 to 2014 after the season to every bobcat hunter who received a tag. A follow-up survey was sent to all non-respondents, with duplicate answers removed from the survey pool. The hunters were asked specific questions about their hunting and trapping methods used during the season. From these surveys we determined the final disposition of the carcass (being sold for fur, being tanned and either kept or sold, or being made into a taxidermy mount) annually by hunting method.

### 2.2. Statistical analyses

We first summarized annual bobcat harvest from 1973 to 2014, which includes data from 5824 bobcats with known age and sex from 1983 to 2014. We used program *R* version 3.3.1 [29] for statistical analyses unless otherwise noted specifically. We considered *p* < 0.05 to be statistically significant. We then used 1800 bobcats from 1983–1999 (when mass data was collected by WDNR) that had known age, sex and mass to test if the mass of harvested bobcats was affected by their sex and age. After testing for normality, we used linear regression to fit models of mass as functions of sex and age. We also tested whether the method of harvest changed over time, to determine if changes to the bobcat population and reducing harvest permits had led to an increase in hunters compared to trappers over time. We used year as our independent variable, substituting an integer for year where 1983 = 1, 1984 = 2, 1985 = 3 and so on. We used our data collected from 1983 to 2014 and fit the data using a binomial generalized linear model (GLM; with trapping = 0 and hunting = 1).

We tested for evidence of harvest selectivity for demographic variables (mass, male:female sex ratio and age) among bobcat hunters compared to trappers. We hypothesized that hunters would select for larger, older, male bobcats and that selectivity would increase over time and with an increasing bobcat population. For each demographic variable, we fit data to a GLM with multiple independent variables, which varied depending on the variable. We used year as our independent variable, substituting an integer for year as explained above. For the demographic variables, we also used a quadratic year, substituting an integer for year where 1983 = 1, 1984 = 4, 1985 = 9 and so on, in order to test for asymptotic trends, but in each model, the quadratic year was not significant and we did not include it in our final models. We first tested for selection of heavier bobcats, by fitting the mass data collected from 1983 to 1999 (the years during which mass data were collected by WDNR) using a Gaussian GLM with an identity link, with sex, year, method of hunting and population index as independent variables. Second, we tested for selection of male bobcats, by fitting the sex ratio data collected from 1983 to 2014 using a binomial GLM with a logit link (with female = 0 and male = 1) with year, method of hunting and population index as independent variables. Third, we tested for selection of older bobcats, by fitting the age data collected from 1983 to 2014 using a gamma GLM with an inverse link, with sex, year, method of hunting and population index as independent variables. Because we used the inverse link, we back-transformed the results to make them more intuitive to understand. Last, we calculated an effect size (*r*) for each significant variable in a given model [30], where greater than 0.10 indicates a small effect size, greater than 0.30 indicates a medium effect size and greater than 0.50 indicates a large effect size.

We calculated odds ratios to determine the effects of method and demography on the final disposition of harvested bobcats. We hypothesized that hunters would be more likely to create taxidermy mounts than trappers and calculated the odds ratio of hunters versus trappers for a final disposition of being made into a taxidermy mount versus other dispositions (having the fur tanned or sold). We also hypothesized that harvested animals with trophy characteristics (older, males) would be more likely to be made into taxidermy mounts. We first calculated the odds ratios of males and females being made into taxidermy for other mounts, first for all harvested bobcats, then separately for hunters and trappers. Second, we split harvested bobcats into two age classes: young (less than 2.5 years old) and old (less than 2.5 years old), then calculated the odds ratios of young and old bobcats being made into taxidermy for other mounts, first for all harvested bobcats, then separately for hunters and trappers.

## 3. Results

The number of bobcats harvested in northern Wisconsin since mandatory registration began in 1973 varied by year and decade (table 1); with a decrease during the 1980s and 1990s associated with increased regulation of the harvest, and then an increase during the 2000s.

**Table 1.** Summary of annual bobcat population estimate, harvest and demographics in Wisconsin 1973–2014. Harvest variables include the number of harvest permits issued, total number of bobcats harvested in the state (including First Nations harvest) and just the state harvest administered through hunting permits by the WDNR. Demographic variables from total harvest include the number of bobcats with known age and sex, as well as the mean and median age and mass, and sex ratio.

year	population estimate	tags issued	total harvest	state harvest	known sex and age	age (mean ± s.e.)	age median	sex ratio (M:F)	mass (mean ± s.e.)	mass median
1980	—	no limit	90	90	0	n.a.	n.a.	n.a.	n.a.	n.a.
1981	—	no limit	208	208	0	n.a.	n.a.	n.a.	n.a.	n.a.
1982	—	no limit	139	139	0	n.a.	n.a.	n.a.	n.a.	n.a.
1983	2031	3214	206	206	84	2.58 ± 0.18	1.5	0.92	7.35 ± 0.07	6.8
1984	1800	3089	260	260	97	2.58 ± 0.24	1.5	1.16	8.17 ± 0.07	7.9
1985	1633	4191	189	189	168	2.29 ± 0.21	1.5	1.07	7.37 ± 0.08	6.75
1986	1630	4064	183	183	158	2.73 ± 0.22	1.5	1.34	7.45 ± 0.07	7.45
1987	1688	5114	247	247	220	2.39 ± 0.20	1.5	1.32	7.75 ± 0.07	7
1988	1875	5285	165	165	127	2.26 ± 0.22	1.5	1.06	7.42 ± 0.07	6.6
1989	1929	5051	136	133	103	2.66 ± 0.24	2.5	1.09	7.72 ± 0.07	7.1
1990	1928	4359	98	98	86	1.93 ± 0.23	1.5	0.98	6.96 ± 0.07	6.9
1991	1967	2358	71	71	57	2.13 ± 0.26	1.5	1.21	8.30 ± 0.07	8.3
1992	2148	2300	217	217	181	2.82 ± 0.22	2.5	1.53	8.90 ± 0.07	9.05
1993	1744	2000	160	158	129	2.73 ± 0.23	2.5	1.18	8.89 ± 0.07	8.5
1994	1891	2000	169	165	153	2.84 ± 0.22	2.5	1.82	9.39 ± 0.08	9.45
1995	1999	2000	111	110	77	3.14 ± 0.27	2.5	1.5	8.98 ± 0.08	8.4
1996	2454	2000	166	162	108	2.69 ± 0.24	2.5	1.37	7.93 ± 0.06	7.6
1997	2649	2000	216	199	139	2.81 ± 0.23	2.5	1.06	8.82 ± 0.06	8.8
1998	2653	1860	194	187	167	2.77 ± 0.22	2.5	1.23	9.15 ± 0.08	8.6
1999	3100	1540	187	181	138	2.83 ± 0.23	2.5	1.58	9.09 ± 0.07	9.15
2000	3217	1490	279	276	171	3.90 ± 0.23	3.5	1.41	n.a.	n.a.
2001	3414	780	152	136	110	2.93 ± 0.24	2.5	1.56	n.a.	n.a.
2002	4052	1330	250	213	161	3.21 ± 0.23	2.5	1.06	n.a.	n.a.
2003	4384	1380	371	332	213	4.04 ± 0.22	3.5	1.61	n.a.	n.a.
2004	4387	1370	362	321	228	3.96 ± 0.22	3.5	1.35	n.a.	n.a.
2005	4439	1540	497	447	379	3.31 ± 0.20	2.5	1.71	n.a.	n.a.
2006	4267	1000	356	277	256	3.64 ± 0.21	3.5	1.53	n.a.	n.a.
2007	4028	1030	477	421	342	3.12 ± 0.20	2.5	1.65	n.a.	n.a.
2008	3281	540	367	298	288	3.12 ± 0.20	2.5	2	n.a.	n.a.
2009	3292	475	271	205	210	3.27 ± 0.21	2.5	2	n.a.	n.a.
2010	2972	455	349	275	215	3.57 ± 0.22	2.5	1.95	n.a.	n.a.
2011	3001	475	357	248	237	3.71 ± 0.21	3.5	1.45	n.a.	n.a.
2012	2541	165	242	98	182	3.94 ± 0.23	3.5	1.59	n.a.	n.a.
2013	2598	215	226	124	136	3.58 ± 0.24	2.5	1.83	n.a.	n.a.
2014	2432	310	264	180	160	3.82 ± 0.23	3.5	1.98	n.a.	n.a.

Mass of harvested bobcats varied significantly by sex (*F*_1_ = 1125.4, *p* < 0.0001) and age (*F*_15_ = 191.1, *p* < 0.0001). Male bobcats were, on average, 2.85 kg heavier than females, while 0.5- and 1.5-year-old bobcats weighed significantly less than bobcats of other ages. On average, male bobcats of age 1.5 and older were heavier than female bobcats of any age (table 2).

**Table 2.** The mass of male and female bobcats (skinned) by age harvested in Wisconsin (1983–1999).

age	0.5	1.5	2.5	3.5	4.5	5.5	6.5	7.5	8.5	9.5+
male										
mean	5.16	10.18	11.48	11.83	12.03	12.72	12.31	12.48	12.86	12.39
s.e.	0.10	0.15	0.16	0.16	0.23	0.25	0.35	0.65	1.07	0.54
median	5.0	10.4	11.4	11.9	12.1	12.8	12.6	12.5	13.9	13.2
low	2.0	3.1	4.3	5.5	6.6	8.1	8.9	7.4	4.1	6.0
high	12.4	16.1	18.4	15.0	15.7	17.3	16.8	16.1	18.0	15.2
count	267	220	154	123	71	52	25	15	11	17
female										
mean	4.35	7.11	7.53	7.97	8.14	8.60	7.97	8.89	8.76	8.79
s.e.	0.07	0.09	0.13	0.16	0.18	0.23	0.24	0.41	0.29	0.33
median	4.30	7.10	7.60	7.95	8.30	8.30	8.20	9.35	8.55	8.65
low	1.3	2.4	3.0	4.6	5.4	6.0	4.3	6.4	7.0	5.7
high	8.5	15.6	14.9	12.2	12.1	12.7	10.2	12.2	10.5	12.6
count	232	222	126	76	57	43	33	16	14	24

**Figure 2.**
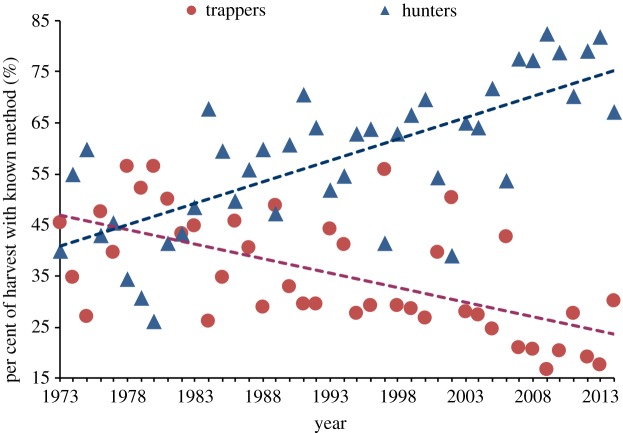
Trends in the annual per cent of bobcat harvest with known method by trappers and hunters in Wisconsin (1973–2014).

Hunting and trapping were the most common methods of harvest, nearly always accounting for over 90% of the annual bobcat harvest with a known method since 1983. Small numbers of bobcats are harvested annually through calling, and some bobcats are taken incidentally to other trapping. The ratio of bobcats harvested by hunters compared to trappers has increased significantly over time (*z*_4,241_ = 7.43, *p* < 0.0001) (figure 2), and quadratic time (*z*_4,241_ = 7.38, *p* < 0.0001).

We found that the effects of year, method of harvest, sex and population varied among demographic variables (table 3). The mass of harvested bobcats was significantly affected by sex (*t*_1261_ = 16.62, *p* < 0.0001, *r* = 0.42), method of harvest (*t*_1261_ = 4.83, *p* < 0.0001, *r* = 0.13) and year (*t*_1261_ = 4.89, *p* < 0.0001, *r* = 0.14), but not population abundance (*t*_1261_ = −1.17, *p* = 0.24). We found the male:female sex ratio of harvested bobcats was significantly affected by method of harvest (*z*_4,213_ = 12.29, *p* < 0.0001, *r* = 0.33) and year (*z*_4,213_ = 3.11, *p* = 0.0004, *r* = 10), but not population (*z*_4,213_ = −1.27, *p* = 0.20). The age of harvested bobcats was significantly affected by sex (*t*_4,035_ = −2.16, *p* = 0.0310, *r* = 0.06), method of harvest (*z*_4,035_ = 2.64, *p* = 0.0084, *r* = 0.07), year (*z*_4,035_ = 7.981, *p* < 0.0001, *r* = 0.22) and population (*z*_4,035_ = 2.97, *p* = 0.0030, *r* = 0.08).

**Table 3.** The results of the generalized linear models (GLMs) testing for harvest selectivity among bobcat demographic variables (mass, age, and sex proportion) in Wisconsin. We list the independent variables, and their mean, s.e., *t* or *z* value, *p* value and effect size (*r*).

variable	estimate	s.e.	*t* value	*p* value	*r*
mass					
(intercept)	5.9973	0.4939	12.14	<0.0001	—
sex	2.6754	0.1610	16.62	<0.0001	0.42
hunting method	0.8002	0.1656	4.83	<0.0001	0.13
year	0.1286	0.0263	4.89	<0.0001	0.14
population size	−0.0004	0.0003	−1.17	0.24	—
sex proportion					
(intercept)	−0.2854	0.1079	−2.64	0.008	—
hunting method	0.8356	0.0680	12.29	<0.0001	0.33
year	0.0166	0.0047	3.51	<0.0001	0.10
population size	−0.0001	0.0000	−1.27	0.20	—
age					
(intercept)	−0.4430	0.0151	−29.35	<0.0001	—
hunting method	0.0227	0.0086	2.64	0.008	0.07
year	0.0042	0.0005	7.98	<0.0001	0.22
sex	−0.0169	0.0078	−2.16	0.03	0.06
population size	−0.0000	0.0000	2.97	0.003	0.08

**Figure 3.**
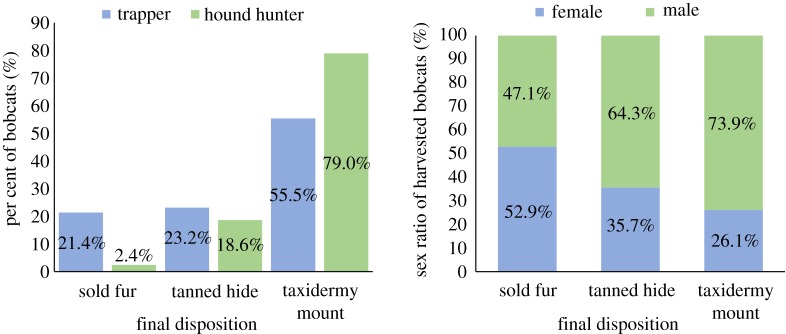
On left, the per cent of the final disposition of bobcats harvested by trappers and hunters. On right, the sex ratio of bobcats harvested by hunters as they correspond to final disposition.

The final disposition of bobcats harvested varied by harvest method (figure 3). Hunters were 3 times more likely to create taxidermy mounts from their harvested bobcats than trappers (odds ratio = 3.01). Male bobcats were almost twice as likely to be made into taxidermy mounts as female (odds ratio = 1.84), with the effect stronger among hunters (odds ratio = 1.69) than trappers (1.27). Older bobcats were slightly more likely to be made into taxidermy mounts than younger bobcats (odds ratio = 1.18), with the effect stronger among trappers (odds ratio = 1.27) than hunters (odds ratio = 1.12).

## 4. Discussion

We analysed dynamics in the harvest demographics of bobcats in Wisconsin over 32 years and found that the changing demographics are partly related to an increasing population, but also more likely due to trends in the selection of (i) larger, (ii) older and (iii) male bobcats by hunters. We found an increase in the proportion of bobcats that were harvested by hunting compared to trapping from 1973 to 2014, and that hunters were more likely to create taxidermy mounts than trappers. The selected traits (males, larger mass and older age) of harvested bobcats were interrelated, as males are usually larger than females and size also increases with age (table 2). From this, we inferred that harvest selection for larger size led to the selection of older male bobcats. This is also supported by other harvested felids also showing similar patterns of older males being selected preferentially [31]. In Wisconsin, there has also been a trend of a decreasing number of harvest permits available over this period (table 1), and these restrictions have potentially led to an increase in the perceived value of the individual bobcat harvested (*sensu* [15]), and an increase in the selection of animals with trophy characteristics.

The selection of older, male bobcats appears primarily driven by hunters compared to trappers, who are increasing in number, can make selection decisions at multiple stages, and were more likely to create taxidermy mounts than to sell the harvested fur. The population of trappers has been ageing and decreasing across North American due to shifting cultural beliefs, reduced economic gain and decreasing access to land for trapping [32–34]. Releasing a trapped bobcat is more difficult than choosing to not harvest a bobcat that has been treed by hounds, hence it is easier for a hunter to be selective. Selection also occurs by hunters at the stage of choosing which individuals to pursue, as hunters can choose to pursue larger animals and male bobcats have larger tracks than females. It is also possible that life-history differences in the sex and age classes of felids may make male bobcats more likely to be found and pursued by hounds [31]. For example, adult male felids, including bobcats, tend to cover larger areas, overlap with multiple females and more often make their presence known through scent marking [20,22,35]. The final disposition of harvested bobcats also varied by the method, with hunters more frequently creating a taxidermy mount, and people who create taxidermy mounts may be more likely to select for trophy characteristics compared to those interested in selling fur for which prime quality is the primary concern. In Wisconsin, hunter selection is compounded by an increasing trend of hunters compared to trappers over the last few decades, leading to what appears to be substantial changes in the demography of harvested bobcats over time.

Preferential selection can have negative effects on wildlife populations over time [8,10,36] and selection exhibited by hunters may bias population models that are based on the demography of harvested bobcats. Preferential selection can bias the sex ratio and age structure of populations [7,8], potentially decrease population growth rate [8] and can alter the social organization of the population [36,37], although male-biased harvest may have a lower demographic impact than random harvest. These patterns are most frequently noted with ungulates (e.g. [7,8]), but could also occur with other hunted species such as furbearers. The restricted harvest of bobcats in Wisconsin may lead to only a small percentage of the population being harvested each year compared to neighbouring states, which could buffer these effects, but the potential negative effects should be considered in other states, provinces or countries, and among species where population-level effects may occur. There has also been increasing harvest success (per cent of filled harvest permits) in Wisconsin over the last few decades, decreasing from annual success rates of 3–8% in the 1980s to annual success rates of over 50% every year since 2008 (table 1). Increased success results in more opportunities for selection, as hunters can pass on multiple treed bobcats before harvesting one. Increased success also often leads to an increased investment and commitment to specialized skills [19,38] such as hunting with hounds. Increased harvest success could therefore be increasing state-wide interest in hunting bobcats with hounds, creating a positive feedback loop and increasing the selection of trophy bobcats over time.

Accounting for biases that may occur, either from a selection of trophy individuals or different harvest methods, is critical when using harvest data-based models. In these models, analysts typically assume the sex and age structure of the harvest to be representative of the population as a whole [4], or that any bias is constant over time [4,39]. Selection for older, male bobcats likely skews the survival estimates and sex-age structure that are integral to harvest-based population models, leading to potential inaccuracies in population estimates. Bobcats harvested by different methods provide different views of the demography of the population in Wisconsin. Bobcats harvested by trappers may be more representative of the population; and if trappers were non-selective, then it would be possible to use the demography of bobcats harvested by trappers for population estimates. Trapping has been declining in Wisconsin, however, and the trapped harvest would currently yield limited sample sizes. Strategies should be considered to determine potential methods to reduce sampling bias in sex and age data from harvested animals.

Bobcat populations are becoming more abundant across North America, including Wisconsin, coincident with decreases in populations of trappers, and this may fundamentally change harvest and demography of harvested bobcats. Increases in bobcat abundance have led to increased densities in prime habitat and also expansion into suboptimal habitat [40]. Decreases in populations of trappers may be compensated for in some states and provinces by increases in harvest by hound hunters. Bobcat harvest in Wisconsin, however, has seen the annual number of harvest permits become much more restrictive [27], and we have shown evidence of selection for older and larger male bobcats. The dynamics of an increasing population and decreasing harvest permits may make Wisconsin unique among states and provinces, or selection by hunters may just be more apparent in Wisconsin due to the extremity of these dynamics. Owing to the potential problems that selection for trophy animals can cause for harvest-based population models, these factors should be considered by scientists when assessing and managing populations across North America.

## Supplementary Material

Data

## Data accessibility

The data used in the analyses for this manuscript is available in electronic supplementary material, S1.
